# Method of Determining Kinetic Parameters of Strength Recovery in Self-Healing Ceramic Composites

**DOI:** 10.3390/ma16114079

**Published:** 2023-05-30

**Authors:** Mostafizur Rahman, Taiyo Maeda, Toshio Osada, Shingo Ozaki

**Affiliations:** 1 Graduate School of Engineering Science, Yokohama National University, Tokiwadai 79-5, Hodogaya-ku, Yokohama 240-8501, Kanagawa, Japan or mostafiz_rasel64@cuet.ac.bd (M.R.); maeda-taiyo-rp@ynu.jp (T.M.); 2 Department of Mechanical Engineering, Chittagong University of Engineering & Technology (CUET), Chattogram 4349, Bangladesh; 3 High Temperature Materials Group, Research Center for Structural Materials, National Institute for Materials Science, Sengen 1-2-1, Tsukuba 305-0047, Ibaraki, Japan; osada.toshio@nims.go.jp; 4 Division of System Research, Faculty of Engineering, Yokohama National University, Tokiwadai 79-5, Hodogaya-ku, Yokohama 240-8501, Kanagawa, Japan

**Keywords:** self-healing ceramics, oxidation kinetics, activation energy, frequency factor, strength recovery

## Abstract

Self-healing ceramic composites are promising smart materials for high-temperature applications. Experimental and numerical studies have been performed to more fully understand their behaviors, and kinetic parameters such as the activation energy and frequency factor have been reported to be indispensable for investigating healing phenomena. This article proposes a method of determining the kinetic parameters of self-healing ceramic composites using the oxidation kinetics model of strength recovery. These parameters are determined by an optimization method using experimental strength recovery data under various healing temperatures, times, and microstructural features on the fractured surfaces. Alumina and mullite matrix-based self-healing ceramic composites such as Al_2_O_3_/SiC, Al_2_O_3_/TiC, Al_2_O_3_/Ti_2_AlC (MAX phase), and mullite/SiC, were selected as the target materials. The theoretical strength recovery behaviors of the cracked specimens obtained from the kinetic parameters were compared with the experimental results. The parameters were within the previously reported ranges, and the predicted strength recovery behaviors reasonably agreed with the experimental values. The proposed method can also be applied to other self-healing ceramics with matrices reinforced with different healing agents to evaluate oxidation rate, crack healing rate, and theoretical strength recovery behaviors to design self-healing materials used in high-temperature applications. Furthermore, the healing ability of composites can be discussed regardless of the type of strength recovery test.

## 1. Introduction

Self-healing ceramics are expected to be used in high-temperature structural applications with high safety requirements owing to their unique autonomic ability to heal surface cracks and their outstanding mechanical properties, such as lightness, high thermal resistance, corrosion resistance, wear resistance, and high fracture strength. Therefore, they have garnered the attention of materials researchers analyzing mechanical behaviors under service conditions aiming to develop outstanding candidates for self-healing ceramics used in sophisticated applications [1,2,3,4,5,6,7,8]. Under such circumstances, experimental and numerical investigations have been conducted to analyze the self-healing capabilities of ceramics and their effects on their mechanical properties.

To the best of the authors’ knowledge, the earliest work related to the so-called “healing” mechanism reported by Heuer et al. [9] in 1966 was based on confirmation of the effect of annealing on the strength of alumina crystals. Lange et al. [10] analyzed the effect of annealing on the crack healing of thermally shocked ZnO and used the term “crack-healing” in 1970. Pioneering work realizing “autonomous” and “full” strength recovery was reported by Ando et al. in 1995 [11], where the oxidation-induced self-healing of cracks was proposed in mullite/SiC ceramic composites. Subsequently, this phenomenon was reported in Si_3_N_4_/SiC ceramic composites in 1998 [12] and 1999 [13], respectively. Later, inspired by the natural self-healing that occurs in the human body, such as that of bones, extensive studies were performed on different matrices and healing agent-based composites such as Al_2_O_3_/SiC [14,15,16,17], Al_2_O_3_/TiC [18], Al_2_O_3_/TiC/TiB_2_ [19], and Al_2_O_3_ with Ni-based nanocomposites [20,21,22], Al_2_O_3_/SiCw [23], Si_3_N_4_/SiC particle [24], Si_3_N_4_/SiCw [25], SiC/SiC ceramics [26], SiN/SiC [27], mullite/SiC [28], mullite/SiC/Y_2_O_3_ [29], ZrO_2_/SiC/TiO_2_ [30], and MAX-phases ceramics [31,32,33,34,35]. Self-crack healing ability and strength recovery behaviors were demonstrated by adopting different strength testing methods. The self-crack-healing ability of high-temperature structural materials under service conditions is an emerging invention to ensure mechanical reliability and integrity in practical applications. The experimental investigations in the aforementioned studies were conducted through variations of the healing temperature, time, environment (N_2_, Ar, vacuum, and mixed O_2_-N_2_), oxygen partial pressure, volume fraction of the healing agents (15–30%), particle size of the healing agents (micro, nano), and indented crack size (40–1200 μm); by a crack indentation method (Vickers, Knoop); by healing under no stress and applied stress conditions; and by utilizing a healed strength testing method (three- and four-point bending). However, experimental investigations are expensive, time-consuming, and require sophisticated experimental setups for the analysis of self-healing ceramic materials before and after healing. 

Meanwhile, novel numerical analysis schemes need to be developed to analyze the behaviors of self-healing ceramics considering real conditions in application fields to ensure reliability, mechanical integrity, and safety margins. Numerical analysis using finite element modeling is a suitable option for the frequent study of self-healing ceramic materials. Continuum damage-healing constitutive models used to analyze the damage formation and healing capability of different self-healing materials have been developed at different times. Under these circumstances, Ozaki et al. [36] successfully developed constitutive equations based on the isotropic damage model [37] and applied them to alumina matrix–based ceramics within the framework of finite element modeling. Subsequently, they developed an oxidation kinetics-based crack healing model and applied it to alumina/SiC-based self-healing composites [38]. More importantly, Osada et al. [39] developed a universal kinetics model for the strength recovery of self-healing ceramics. In this model, microstructural information, crack geometry, the volume fraction of healing agents, crack propagation direction, and kinetic parameters of healing agent oxidation were considered. They also proposed a tip-to-mouth crack-gap filling model that could be applied to different matrix-based composites by incorporating different healing agents. However, no framework is available to determine the kinetic parameters, such as the activation energy (AE) and frequency factor (FF) of healing-agent oxidation in self-healing ceramic composites. To understand the self-healing capability under service conditions more fully, the determination of AE and FF is indispensable.

Furthermore, once the AE and FF are determined, they can be applied to finite element analysis as parameters of the damage-healing constitutive model [38]. In addition, the full experimental datasets of temperature- and time-dependence of the minimum healing time for the full strength recovery and strength recovery rate required for healing ability comparison of the above self-healing ceramics were limited in the previous studies because different researchers tested different materials under various experimental conditions, as shown in Table 1. A framework determining those kinetic parameters from limited experimental data could be very useful for new material design.

In this study, we developed a novel method to simultaneously determine these kinetic parameters by optimizing a universal kinetics model, using experimental data on strength recovery behaviors. This method can predict the suitable range of kinetic parameters for each experimental set of composites by performing multiple analyses of the healing conditions. In addition, the theoretical strength recovery behaviors of several self-healing ceramic composites were predicted using these kinetic parameters, and the effectiveness of the optimized kinetic parameters was confirmed through comparison with previously reported experimental results. This study primarily focuses on determining the AE and FF, and the characteristics of the oxidation rate, healing rate, and minimum time required for complete healing of different healing agents incorporated with alumina and mullite matrices. The oxidation and healing rates of healing agents with the matrix, which are necessary for the selection of suitable candidates for applications, can be predicted using these parameters.

## 2. Universal Kinetic Model of Strength Recovery

This section describes in detail the volume-gain kinetics model and tip-to-mouth crack-gap filling models for self-healing ceramic materials [39] underlying the parameter determination method.

### 2.1. Volume-Gain Kinetics Model

**Figure 1:**
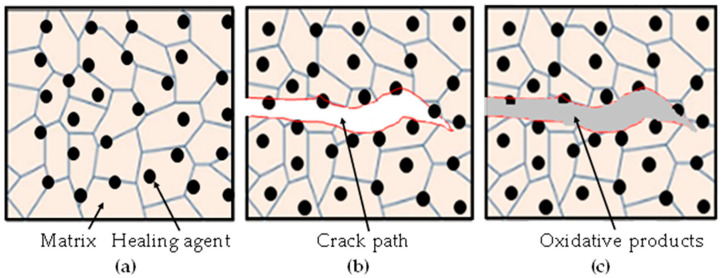
Schematic illustration of crack-gap filling by oxidative products produced from the oxidation reaction of the healing agents incorporated within the matrix: (**a**) healing agents are located within the matrix such that micro-sized healing agents are located on the grain boundary and nano-sized healing agents on the grain structure; (**b**) crack initiation, propagation direction both along the interface of the matrix-healing agents and through the healing agents, where this propagation direction depends on the mechanical properties between the matrix and healing agents; (**c**) crack-gap filling by oxidative products, which could be complete or partial depending on the healing conditions.

The healing mechanisms of self-healing ceramics are explained by an oxidation kinetics-based healing model [39]. This model can be applied to various healing agent-based composites. Volume gains due to oxidation-induced reaction lead to weight gains, and a schematic illustration of this phenomenon is presented in Figure 1.

The isothermal weight gain formula can be expressed as a function of the healing temperature and time, oxygen partial pressure, kinetics parameters, and features of the fractured surface and healing agents. The general formulation of the isothermal weight gain is as follows:

(1) $$∆w={\left(k_{p}∆t_{H}\right)}^{\frac{1}{n}},$$

where $k_{p}$ is the oxidation rate constant, $∆t_{H}$ is the holding healing time, and *n* signifies the rate-controlling oxidation mechanism. For the oxidation of SiC as a healing agent, *n* = 2 can be adopted [39]. Meanwhile, *n* = 2 was also considered for the TiC healing agent oxidation. Furthermore, *n* = 3 was adopted for the oxidation of Ti_2_AlC MAX-phase healing agent in an alumina matrix [35]. The rate constant $k_{p}$ can be formulated by considering the effect of the oxygen partial pressure as follows:

(2) $$k_{p}=k_{p}^{o}\;\exp\left(-\frac{Q_{ox}}{RT_{H}}\right){\left(\frac{P_{O_{2}}}{P_{O_{2}}^{0}}\right)}^{m},$$

where $Q_{ox}$ and $k_{p}^{o}$ are AE and FF of healing agent-oxidation, respectively, for the oxidation of the healing agents; *T_H_* is the healing temperature; $P_{O_{2}}$ is the oxygen partial pressure; $P_{O_{2}}^{0}$ is the standard oxygen partial pressure; *R* is the gas constant; and *m* = 0.835 [39] is a temperature-independent constant experimentally determined for the healing reaction in the N_2_-O_2_ mixed gas. Hence, the weight gain from healing-agent oxidation can be described as

(3) $$∆w={\left\{k_{p}^{o}exp\left(-\frac{Q_{ox}}{RT_{h}}\right){\left(\frac{P_{O_{2}}}{P_{O_{2}}^{0}}\right)}^{m}∆t_{H}\right\}}^{\frac{1}{n}}.$$

Furthermore, the volume gain $V_{h}$ during the isothermal oxidation reaction can be converted from the weight gain as follows:

(4) $$V_{h}=\frac{A_{0}}{∆\rho }∆w=\frac{2Af_{v}f_{e}}{∆\rho }{\left\{k_{p}^{o}exp\left(-\frac{Q_{ox}}{RT_{h}}\right){\left(\frac{P_{O_{2}}}{P_{O_{2}}^{0}}\right)}^{m}∆t_{H}\right\}}^{\frac{1}{n}},$$

where $A_{0}$ (reactive area fraction) = 2*A*$f_{v}f_{e},$ and *A* is the area of one side of the fractured surfaces. ∆ρ is the weight gain per unit volume gain for healing agent oxidation. The volume gain owing to the self-healing reaction depends on the free surface of the unreacted healing agents on the fractured surfaces. The volume fraction $f_{v}$ of the healing agents and the crack propagation path determine the actual reactive area fraction of the healing agents. In general, the volume fraction of the healing agents, $f_{v}$, ranging from 0.15 to 0.30, has been used for the complete healing of cracks based on numerous experimental analyses reported in [14,15,28,45]. The effective reactive area ratio, $f_{e}$ is considered to be 0.5 when the crack propagates along the interface of the matrix and healing agents, as the healing agents are located on one side of the fractured surface, as shown in Figure 1. In this study, $f_{e}$ was taken to be 0.5 for SiC healing agent oxidation with an alumina and mullite-based matrix. Furthermore, $f_{e}$ = 1.0 when the crack propagates through the healing agents, which is considered for monolithic ceramics mainly consisting of healing agents, such as SiC and Ti_2_AlC. Notably, $f_{e}$ = 0.5 and $f_{e}$ = 1.0 are assumed in the cases of TiC and Ti_2_AlC oxidation, respectively, as detected from the scanning electron microscope (SEM) images after cracking, which implies that cracks propagate along the interface of the matrix and healing agents and through the healing agents, respectively [43,44]. Here, the crack propagation path could mainly depend on the differences in fracture strength, toughness, and stiffness between the matrix and healing agents [39].

∆ρ is the weight gain per unit volume gain for healing agent oxidation. For SiC oxidation,

(5) $$∆\rho =\frac{M_{SiO_{2}}-M_{SiC}}{\left(\frac{M_{SiO_{2}}}{\rho_{SiO_{2}}}-\frac{M_{SiC}}{\rho_{SiC}}\right)},$$

where *M*_SiC_, *M*_SiO2_, $\rho_{SiC}$, and $\rho_{SiO_{2}}$ are the molar mass and molar density of SiC and SiO_2_, respectively. Furthermore, ∆ρ can be calculated in a similar manner for TiC and Ti_2_AlC oxidation by applying the following general formula:

(6) $$∆\rho =\frac{\left(\sum_{i=1}n_{i}M_{HP}^{i}-M_{HA}\right)}{\left(\sum_{i=1}\frac{n_{i}M_{HP}^{i}}{\rho_{HP}^{i}}-\frac{M_{HA}}{\rho_{HA}}\right)},$$

where $M_{HP}^{i}\;\mathrm{and}\;\rho_{HP}^{i}$ are the molar mass and molar density of the *i*th healing product (HP), respectively, and $M_{HA}$ and $\rho_{HA}$ are the molar mass and molar density of healing agents, respectively. In this study, ∆ρ = 1.4908 × 10^3^, 2.9760 × 10^3^, and 4.7133 × 10^3^ were used for SiC, TiC, and Ti_2_AlC healing agent oxidation, respectively.

Finally, the volume-gain rate from the oxidation kinetics model can be expressed as follows:

(7) $$\dot{V_{h}}=\frac{1}{nV_{h}^{n-1}}{\left(\frac{2Af_{v}f_{e}}{∆\rho }\right)}^{n}k_{p}.$$

The initial value of volume gain $V_{h}^{0}$ in the numerical analysis can be written as follows:

(8) $$V_{h}^{0}={\left\{{\left(\frac{2Af_{v}f_{e}}{∆\rho }\right)}^{n}k_{p}{∆t}_{0}\right\}}^{\frac{1}{n}}.$$

Here, ${∆t}_{0}$ is the initial time increment. Equations (4), (7) and (8) are useful for determining the healing parameters of different healing agents under isothermal and non-isothermal conditions.

### 2.2. Tip-To-Mouth Crack-Gap Filling Model

**Figure 2:**
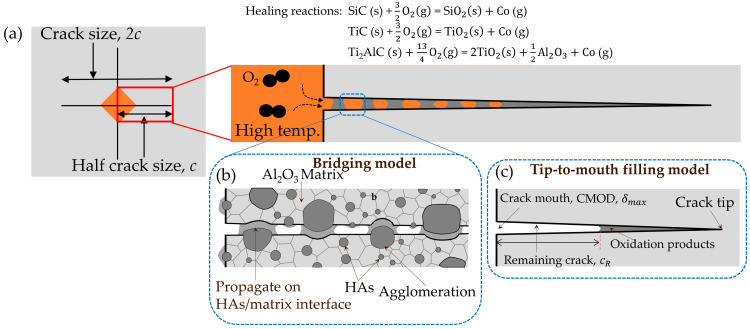
Schematic illustration of tip-to-mouth crack-gap filling by oxidation products produced by healing agents. SiO_2_, TiO_2_, and Al_2_O_3_ fill the crack gap and make a strong bonding with the matrix using the heat produced by these exothermic healing reactions. The remaining crack length on the surface, $c_{R},as$ calculated from Equation (14), measures the level of the cracked strength recovery: (**a**) crack indentation, geometry, and crack-gap filling; (**b**) crack-gap filling by bridging model; (**c**) crack-gap filling by tip-to-mouth model.

Various experimental studies have been conducted on the self-healing of cracks induced by the Vickers indentation method. Many researchers have revealed that these indented cracks are completely or partially healed with different healing temperatures, times, and other parameters [15,17,23]. The experimental strength of the cracked specimens is recovered through crack-gap filling by the oxidation products from the healing agents incorporated into the matrix. Note that crack-gap filling can be modeled theoretically using the tip-to-mouth crack-gap filling model and bridging model [39]. In this study, the tip-to-mouth crack-gap filling model was adopted. The crack-gap filling mechanism in ceramics and composites includes chemical reactions between the healing agents and surrounding oxygen to produce oxidative products, as shown in Figure 2. Osada et al. [41] demonstrated for alumina/SiC composites that these chemical reactions comprise three steps: inflammation, repair, and remodeling. The healing agents react with the surrounding oxygen to produce reactive products in the inflammation step, and these products react with the matrices to generate supercooled melt, leading to full crack gap filling. Finally, the supercooled melt causes crystallization at the crack locations.

The crack-gap filling ratio $R_{f}$ is an important parameter that determines the level of strength recovery during self-healing and can be written as follows:

(9) $$R_{f}=\frac{V_{h}}{V_{g}}.$$

Here, $V_{g}$ is the crack-gap volume and can be expressed as follows:

(10) $$V_{g}=\frac{\pi \delta_{max}}{6}(c^{2}-ac+2a^{2}),$$

where c is the half-crack length, as shown in Figure 2a, and a is the crack depth of a median/radial crack introduced by Vickers indentation and is defined by the aspect ratio a/c = 0.9. $\delta_{max}$ is the maximum crack mouth opening displacement (CMOD), which is related to the initial CMOD, and decreases significantly due to annealing. In this study, we adopted the following empirical equations applicable to alumina matrix-based composites [39] to evaluate $\delta_{max}$:

(11) $$\delta_{max}=\delta_{max}^{0}\exp\left\{-{\left(\frac{T_{H}}{T_{b}}\right)}^{q}\right\},$$

where $\delta_{max}^{0}$ is the initial CMOD before annealing. $T_{b}$ = 1523 K and q = 9 are the empirical fitting parameters. The empirical relation between $\delta_{max}^{0}$ and half-crack size *c* can be expressed as follows:

(12) $$\delta_{max}^{0}=4.06\;c.$$

The total crack-gap volume after annealing for a specific crack geometry induced by Vickers indentation can be estimated using Equations (10)–(12). Hence, the filling ratio $R_{f}$ for SiC and TiC healing agent oxidation when incorporated within alumina matrix-based composites becomes

(13) $$R_{f}=\frac{\frac{2Af_{v}f_{e}}{∆\rho }\sqrt{\left\{k_{p}^{o}exp\left(-\frac{Q_{ox}}{RT_{h}}\right){\left(\frac{P_{O_{2}}}{P_{O_{2}}^{0}}\right)}^{m}∆t_{H}\right\}}}{\frac{\pi \delta_{max}}{6}(c^{2}-ac+2a^{2})},$$

where one side area of the fractured surface is expressed as *A* = $\frac{\pi }{2}$($c^{2}-a^{2}$). As assumed, the oxidation products start filling the crack from the crack tip point to the mouth in the tip-to-mouth filling model. Furthermore, the crack opening displacement increases linearly from the tip to the mouth, as shown in Figure 2a. Thus, the crack size evolution in this model is given by

(14) $$c_{R}=c(1-\sqrt{R_{f}}).$$

It is noteworthy that the strength recovery rate is strongly dependent on the remaining crack length of the surface, $c_{R}$. The theoretical strength recovery rate can be estimated based on nonlinear fracture mechanics [23,46,47] as a function of the remaining crack length on the surface, fracture toughness, and smooth specimen strength:

(15) $$\sigma_{f}={cos}^{-1}\left\{\frac{8c_{R}F^{2}\sigma_{0}^{2}}{\pi K_{IC}^{2}+8c_{R}F^{2}\sigma_{0}^{2}}\right\}\frac{2\sigma_{0}}{\pi },$$

where *F* is a geometric factor that can be estimated using the Newman–Raju equation [48]. For simplicity in the calculation, we used the crack size evolution as determined by Equation (14) and the geometric factor estimated by the Newman–Raju equation. $\sigma_{0}$ is the strength of smooth specimen. *K_IC_* is the mode I fracture toughness of the composite, which can be expressed as follows:

(16) $$K_{IC}=K+K_{res},$$

where *K* is the stress intensity factor at the crack tip caused by the external applied stress and $K_{res}$ is the stress intensity factor at the crack tip caused by the internal tensile residual stress near the indentation. According to Osada et al. [39], $K_{res}$ can be approximated using the following empirical equation:

(17) $$K_{res}=0.4\;K\;\exp\left\{{-\left(\frac{T_{H}}{T_{c}}\right)}^{p}\right\},$$

where $T_{c}$ = 1423 K and *p* = 11 are the fitting parameters used in this model.

The calculation of fracture stress using Equation (15) was performed by inputting the fracture toughness values obtained from experiments conducted at room temperature in air for each composite. Therefore, the influence of the humidity in a room temperature atmosphere was reflected implicity, although it is considered to be small. The stress intensity factor due to the residual stress is relaxed significantly by high-temperature healing, which is followed in this model to predict the theoretical strength recovery behaviors. The remaining crack size $c_{R}$ in the tip-to-mouth filling model is the only indicator of cracked strength recovery in Equation (15). $R_{f}$ < 1 owing to a low temperature or short healing period corresponds to a partial crack healing state. In contrast, when $R_{f}$ = 1, corresponding to a high temperature or long period of healing, then $c_{R}$ = 0, signifying that complete crack healing leads to full-strength recovery at the same level as the strength $\sigma_{0}$ of the smooth specimen.

## 3. Parameter Determination Method

### 3.1. Target Composites

In this study, alumina matrices reinforced with SiC, TiC, ${Ti}_{2}$AlC, and mullite reinforced with SiC were selected as they are commonly used as composites to investigate the self-crack healing phenomenon under high-temperature conditions. Furthermore, because we adopted the tip-to-mouth crack-gap filling model in this study, which is applicable to alumina- and mullite-based composites and preliminary, three healing agents (SiC, TiC, and Ti_2_AlC) with alumina and mullite matrices were selected to investigate their kinetic parameters, healing agent-oxidation rates, and average strength recovery rates. In these composites, the volume fraction of healing agents was designed to be 15–30%, according to the healing kinetics requirement, to achieve strength recovery. The alumina reinforced with 15 and 30 vol.% SiC was referred to as “AS15P” and “AS30P,” respectively, and mullite reinforced with 20 vol.% SiC was referred to as “MS20P,” and they were treated as SiC healing agent-based composites to investigate their oxidation and healing capabilities.

The alumina reinforced with 15 and 30 vol.% TiC belongs to TiC healing agent-based composites that are among the emerging self-healing ceramic candidates, as they can be used in applications with comparatively low healing temperatures. We refer to these composites as ‘AT15P’ and ‘AT30P’, respectively. In addition, the alumina matrix reinforced with a MAX-phase healing agent (${Ti}_{2}$AlC) is another candidate for autonomous crack healing in high-temperature applications. Notably, MAX-phase ceramics act as both matrices and healing agents owing to their physio-chemical natures [44]. Alumina reinforced with 20 vol.% ${Ti}_{2}$AlC, referred to as “AT20MAX,” belongs to MAX-phase healing agent-based composite.

The parameter determination method utilizes the strength recovery behaviors corresponding to healing conditions, micro-structural information, crack geometries, and other experimental data. The basic information about the target composites is summarized in Table 1. The experimental healing process consists of three steps: heating, holding, and cooling. Although the healing temperature and time in the holding stage are crucial for healing and cracked strength recovery, the heating and cooling stages cannot be ignored in the healing behaviors. These stages are strongly reflected in the parameter determination and theoretical strength recovery calculations.

### 3.2. Parameter Optimization

The determination of the AE and FF of healing agent oxidation based on optimization is the most significant aspect of this study. The AE and FF are optimized by comparing the experimental and theoretical strength recoveries corresponding to the healing temperature, time, and other features. Here, the theoretical strength recovery calculated using Equation (15) strongly depends on the remaining crack size obtained using the tip-to-mouth crack-gap filling model. Therefore, micro-structural features such as crack geometry and its aspect ratio, size, and volume percentage fraction of the healing agents are used to calculate the theoretical strength recovery.

In the parameter optimization, thousands of combinations of AE and FF were automatically varied within the prescribed range with a small interval in the theoretical calculations to reduce the absolute error (the error between the experimental and theoretical strengths) while keeping the other parameters fixed. Here, the lowest absolute error point between the theoretical and experimental strengths was explored using Equations (18) and (19). In general, the mean relative error and least squares method are used to optimize different scientific parameters by calculating the absolute error.

Firstly, the mean relative error in this study can be calculated as follows:

(18) $$\mathrm{mean}\;\mathrm{relative}\;\mathrm{error}=\frac{1}{N}\left[\sum_{i=1}^{nn}\sum_{j=1}^{mm}\frac{\left|\sigma_{\exp\left(ij\right)}-\sigma_{th(ij)}\right|}{\sigma_{\exp\left(ij\right)}}\right],$$

where *i* = 1 to *nn* represents different healing temperatures and *j* = 1 to *mm* represents different healing times. $\sigma_{exp(ij)}$ is the experimental strength corresponding to the healing temperature and time, whereas $\sigma_{th(ij)}$ is the theoretical strength corresponding to the experimental conditions. *N* is the total number of healed strength points considered in the optimization. The mean relative errors were plotted on the global and local error curves to explore the optimized points of AE and FF. The proposed method provides the AE and FF values corresponding to the lowest error, which is considered the best combination of the AE and FF for a target self-healing ceramic composite.

Furthermore, the least squares method was applied to calculate the error to optimize the AE and FF. The mean squared error can be written as follows:

(19) $$\mathrm{mean}\;\mathrm{squared}\;\mathrm{error}=\frac{1}{N}\left[\sum_{i=1}^{nn}\sum_{j=1}^{mm}{\left(\sigma_{\exp\left(ij\right)}-\sigma_{th(ij)}\right)}^{2}\right].$$

The relative error method and least squares method based on Equations (18) and (19) can be used to explore the AE and FF for every ceramic composite, and they output almost the same AE and FF values. Therefore, this article reports only the AE and FF values obtained from the relative error method, which were utilized to calculate the theoretical strength recovery, as described in Section 4. Suitable ranges of the AE and FF for all composites can be obtained by considering multiple experimental healed strength data points, including completely healed and partially healed strengths.

## 4. Results

The theoretical strength recovery behaviors shown in this section for all composites use the optimized AE and FF values. It should be noted that the experimental healed strength data above the bold lines in the graphs were not considered in the optimization because the fracture started from outside the healed part in the case of super healing.

### 4.1. SiC—Healing Agent-Based Alumina and Mullite Composites

**Figure 3:**
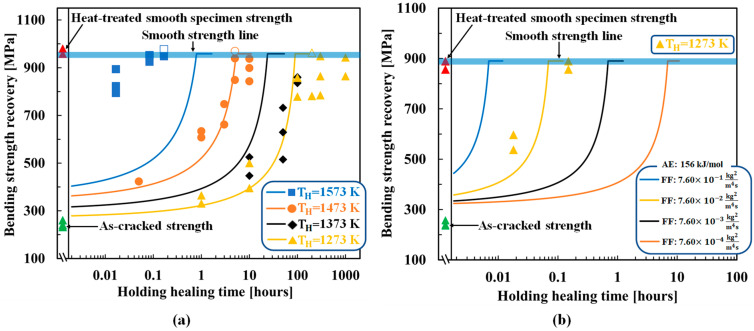
Comparison of strength recovery behavior between the experimental and theoretical results: (**a**) AS30P [39], (**b**) AS30P0.2MnO [41]. The smooth lines correspond to the theoretical strength recovery using the determined AE and FF. The different shaped plots correspond to the experimental strength recovery with different holding healing temperatures and times. The open plots represent the super healing conditions and were not used to determine the AE and FF. “Smooth specimen strength” refers to the “original strength of the sample” (without healing).

Figure 3a compares the experimental and theoretical (Equation (15)) results for the healing temperature- and time-dependent strength recovery behaviors of AS30P. The figure also shows the experimental as-cracked strength and heat-treated smooth specimen strengths. In AS30P, the optimized AE and FF are 136 kJ/mol and 8.90 × $10^{-6}\frac{{kg}^{2}}{m^{4}s}$, respectively. Because the tip-to-mouth filling model corresponds to a lower healed specimen strength [39], the smooth specimen strengths represented by bold lines were adopted for $\sigma_{0}$. The theoretical strength calculated from Equation (15) gradually increased from the as-cracked strength with the holding healing time and reached the strength of the smooth specimen, $\sigma_{0}$, when the remaining crack length $c_{R}$ calculated from Equation (14) became zero ($R_{f}=1$). The holding healing time at which the theoretical strength equals $\sigma_{0}$ is termed the theoretical minimum time for complete healing or full-strength recovery. Even after this time, the theoretical strength lines (smooth lines) remain constant in the figures. The theoretical strength recovery behaviors are in reasonable agreement with the experimental results, except at the high temperature of 1573 K. The error at high temperatures is due to the sensitivity of the initial CMOD to the crack-gap volume calculation in the tip-to-mouth filling model. In addition, the existence of small defects and internal pores around the Vickers cracks was not considered in the theoretical analysis, which is another reason for the difference between the experimental and theoretical cracked strengths.

Figure 3b shows the optimized results for AS30P with the healing activator. In this composite, a healing activator, 0.2 vol.% MnO, was added along with AS30P (referred to as AS30P0.2MnO). The optimized AE and FF were 156 kJ/mol and 7.60 × $10^{-2}\frac{{kg}^{2}}{m^{4}s}$, respectively. Although the AE value was almost identical to that of AS30P, this composite recovers the cracked strength owing to the substantially reduced holding healing time, even at 1273 K. The high FF of 7.60 × $10^{-2}\frac{{kg}^{2}}{m^{4}s}$ caused rapid strength recovery with a higher oxidation capability at 1273 K within a reasonable holding healing time. To demonstrate the effectiveness of the optimized parameters, three other FF values were considered, assuming the same AE of 156 kJ/mol, to compare with the theoretical behaviors. An FF value of 7.60 × $10^{-2}\frac{{kg}^{2}}{m^{4}s}$ yielded better agreement with the experimental data: approximately 6000 times the rapid strength recovery compared to that of AS30P without a healing activator, as reported by Osada et al. [41].

**Figure 4:**
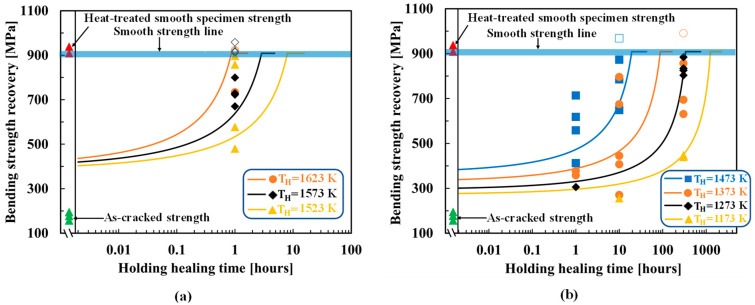
Comparison of the strength recovery behavior of AS15P [40] between the experimental and theoretical results: (**a**) temperature from 1523 K to 1623 K; (**b**) temperature from 1173 K to 1473 K. The smooth lines correspond to the theoretical strength recovery using the determined AE and FF values. The different shaped plots correspond to the experimental strength recovery with different holding healing temperatures and times. The open plots represent the super healing conditions, which were not used to determine the AE and FF. “Smooth specimen strength” refers to the “original strength of the sample” (without healing).

**Figure 5:**
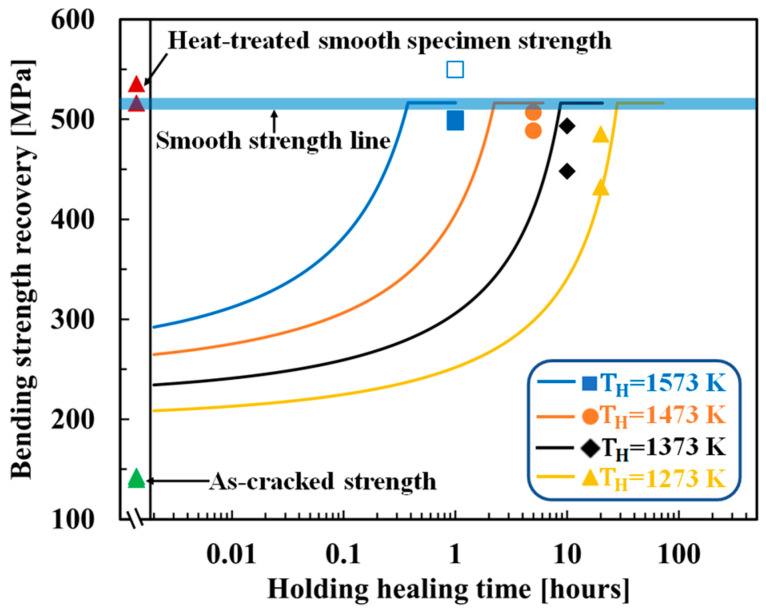
Comparison of strength recovery behavior of MS20P [42] between the experimental and theoretical results. The smooth lines correspond to the theoretical strength recovery using the determined AE and FF values. The differently shaped plots correspond to the experimental strength recovery with different holding healing temperatures and times. The open plots represent the super healing conditions and were not used to determine the AE and FF. “Smooth specimen strength” refers to the “original strength of the sample” (without healing).

Figure 4 shows the optimized results for AS15P. Here, the healing temperatures of AS15P ranged from 1173 K to 1623 K and are separated into high-temperature (1523–1623 K) and low-temperature (1173–1473 K) groups for a clear comparison of the experimental and theoretical strength recovery behaviors. AS15P output an AE of 138 kJ/mol and an FF of 9.50 × $10^{-6}\frac{{kg}^{2}}{m^{4}s}$, exhibiting reasonable agreement between the experimental and theoretical strength recovery behaviors. The optimized results for MS20P are shown in Figure 5. MS20P output an AE of 113 kJ/mol and an FF of 6.0 × $10^{-6}\frac{{kg}^{2}}{m^{4}s}$, which again demonstrates agreement between the experimental and theoretical strength recovery behaviors.

### 4.2. TiC—Healing Agent-Based Alumina Composite

**Figure 6:**
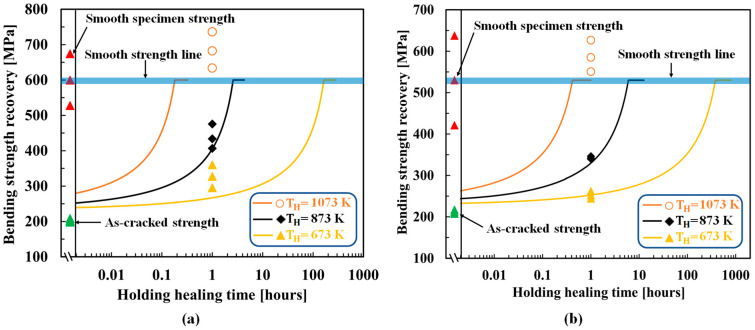
Comparison of strength recovery behavior of TiC healing agent-based alumina composite [43]: (**a**) AT30P; (**b**) AT15P. The smooth lines correspond to the theoretical strength recovery using the AE and FF values determined from the tip-to-mouth filling model. The differently shaped plots correspond to the experimental strength recovery with different holding healing temperatures and times. The open plots represent the super healing conditions and were not used to determine the AE and FF. Here, the middle data is used as the strength of the smooth specimen, $\sigma_{0}$. This method also yields almost the same AE and FF if most of the upper point is used as the smooth specimen strength, $\sigma_{0}$. “Smooth specimen strength” refers to the “original strength of the sample” (without healing).

Yoshioka et al. [43] showed that complete strength recovery in TiC healing agent-based alumina composites and even more than the smooth specimen strength can be achieved at 1073 K/1 h/air, which signifies that TiC oxidizes at a low AE and high FF compared with SiC. Figure 6 shows the optimized results for AT30P and AT15P. The figure also shows the experimental as-cracked strength and heat-treated smooth specimen strength. Because this composite exhibits a large scatter of strength, the strength of the smooth specimen represented by bold lines was adopted for $\sigma_{0}$. TiC exhibits a higher oxidation capability as the as-cracked strength of AT30P and AT15P recovers to the smooth strength under a lower healing temperature and time, as shown in Figure 6. Here, crack propagation along the matrix–healing agent interface is considered owing to the confusion regarding the crack propagation direction from the SEM images reported in [43].

Interestingly, AT30P and AT15P output the same AE of 101 kJ/mol and almost the same FF of 5.20 × $10^{-3}\frac{{kg}^{2}}{m^{4}s}$ and 9.0 × $10^{-3}\frac{{kg}^{2}}{m^{4}s}$, respectively, if cracks propagate along the matrix–healing agent interface. The optimized parameters exhibited suitable agreement between the experimental and theoretical strength recovery behaviors, as shown in Figure 6.

### 4.3. MAX-Phase Healing Agent-Based Alumina Composite

**Figure 7:**
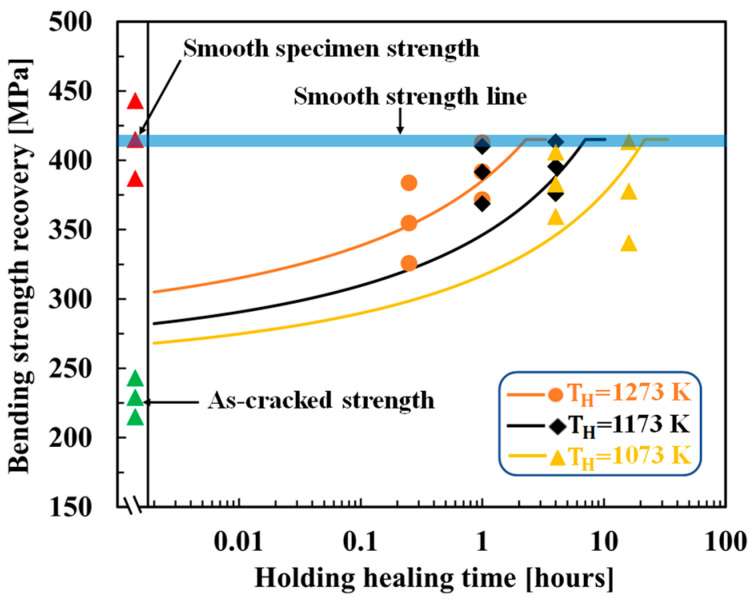
Comparison of strength recovery behavior of AMAX20P [44] between the experimental and theoretical results. The smooth lines correspond to the theoretical strength recovery using the AE and FF values determined from the tip-to-mouth filling model. The differently shaped plots correspond to the experimental strength recovery with different holding healing temperatures and times. Here, the middle data is used as the strength of the smooth specimen, $\sigma_{0}$. “Smooth specimen strength” refers to the “original strength of the sample” (without healing).

Boatemaa et al. [44] analyzed the autonomous surface crack healing of alumina reinforced with 20 vol.% ${Ti}_{2}$AlC and found the strength to be completely recovered at healing temperatures ranging from 1073 K to 1273 K for times of 0.2–16 h. Figure 7 shows the optimized results for AMAX20P, as well as the experimental as-cracked strength and heat-treated smooth specimen strength. Because this composite also exhibited a large scatter of strength, the strength of the smooth specimen represented by bold lines was adopted for $\sigma_{0}$. In this composite, crack propagation through the healing agent was considered for ${Ti}_{2}$AlC oxidation, and a reasonable agreement was observed between the experimental and theoretical strength recovery behaviors. AMAX20P outputs an AE of 101 kJ/mol and FF of 1.80 × $10^{-7}\frac{{kg}^{2}}{m^{4}s}$, if cracks propagate through the healing agent.

It should be noted that some discrepancy exists between the theoretical and experimental strength recovery behaviors in Figure 3, Figure 4, Figure 5, Figure 6 and Figure 7. This difference could be improved by applying not only the tip-to-mouth filling model but also the bridging model (Figure 2). Changes in the geometric factor *F* may also need to be considered when the cracks are almost completely healed [39]. Furthermore, this discrepancy could be reduced by enriching the experimental data set for each composite considered in this study.

## 5. Discussion

**Figure 8:**
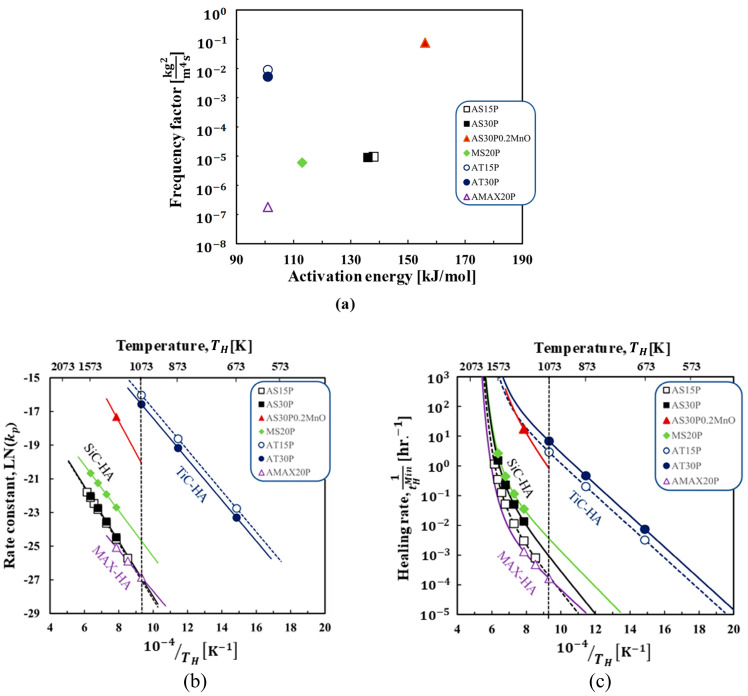
Summary of the determined kinetic parameters of self-healing ceramic composites: (**a**) determined AE and FF values for healing agent oxidation; (**b**) Arrhenius plot of oxidation rate; (**c**) Arrhenius plot of average strength recovery rate. Here, the oxidation and healing rate were calculated using the AE and FF values determined by considering the same damage size (2*c* = 100 μm), *T_H_* is the holding healing temperature of the composites in detail, AS15P $\left(1173–1623\;K\right),$ AS30P $\left(1273–1573\;K\right),AS30P0.2MnO\;(1273\;K)$ MS20P $\left(1273–1573\;K\right),$ AT30P and AT15P $\left(673–1073\;K\right),$ AMAX20P $\left(1073–1273\;K\right)$. The lines are the calculated values, and the plots are the optimization points corresponding to the experimental values.

Figure 8a–c summarizes the determined AE and FF values of healing agent oxidation, the Arrhenius plot of the determined healing agents-oxidation rate for the self-healing ceramic composites, and the Arrhenius plot of the average strength recovery rate, respectively. As shown in Figure 8a, the AE and FF for healing agent-oxidation determined from AS15P exhibit values similar to those of AS30P, despite the different $f_{v}$ values of the healing agents. Further, the AE and FF for AT15P also show almost the same values compared to AT30P. This finding implies that the kinetics model mentioned in Section 2 can accurately describe the effect of the $f_{v}$ of the healing agents on the strength recovery by self-healing. In the SiC–healing agent-based alumina and mullite composites, the AE of SiC oxidation is different in the case of alumina and mullite matrices, despite the same SiC-type healing agent. Further, AS30P0.2MnO shows considerably larger values of FF than the SiC healing agent-based alumina and mullite composites. The AE range for these SiC-healing-based alumina and mullite composites lies within the reported value for SiC oxidation, 84–498 kJ/mol [49], which is reasonable for SiC oxidation. The TiC healing agent-based alumina composite exhibits a lower AE and higher FF than the other composites. Meanwhile, the FF of the ${Ti}_{2}$AlC–healing agent-based alumina composite is lower than those of the SiC–healing agent-based alumina, mullite, and TiC–healing agent-based alumina composites, whereas the AE is lower than those of the SiC–healing agent-based composites.

Further, an Arrhenius plot of the rate constant *kp* of the healing agent oxidation, calculated by applying Equation (2) imputing the determined AE and FF in Figure 8a, is shown in Figure 8b. For the TiC-, SiC-, and MAX-healing agents, a linear plot can be calculated for the $k_{p}$ value of the healing agent oxidation. The figure clearly shows that the values of $k_{p}$ follow the order of TiC-healing agent > SiC-healing agent (MnO) >> SiC-healing agent (mullite) > SiC-healing agent (alumina)~MAX-healing agent. In addition, it is worth mentioning that the $k_{p}$ value of the SiC oxidation is different for the cases of alumina and mullite matrices, despite the same SiC-type healing agent. Furthermore, large values of $k_{p}$ for the healing agent oxidation can be realized by the MnO healing activator addition. This result can be explained by the dissolution of the matrix or healing activator into SiO_2_ formed by SiC-healing agent-oxidation during the “repair stage”, as reported in [41]. This dissolution during the repair stage can decrease the glass transition temperature and viscosity of the healing product, resulting in rapid diffusion of O_2_ and the volume gain $V_{h}$ in Equation (4).

As shown in Figure 8c, the average strength recovery rate $\bar{R_{s}},$ calculated by applying Equation (20) [39] using the AE and FF values determined from Figure 8a, enables comparison of the self-healing ability for all composites under the same damage size (the crack size 2*c* is 100 μm in Figure 2a). This is helpful for designing self-healing ceramic composites. Here, the average strength recovery rate can be calculated from the minimum time required for complete strength recovery in the case of isothermal oxidation [15,39] as follows:

(20) $$\bar{R_{s}}=1/t_{H}^{Min}={\left(\frac{2Af_{v}f_{e}}{∆\rho V_{g}}\right)}^{2}k_{p}^{0}exp\left(\frac{{-Q}_{ox}}{RT_{H}}\right){\left(\frac{P_{O2}}{P_{O2}^{0}}\right)}^{m}.$$

If the crack geometry (*A*, *V_g_*), volume fraction of the healing agents (*fv*), crack propagation path (*f_e_*), weight gain per unit volume gain (∆ρ), and healing agent oxidation rate are known, then the minimum healing time ($t_{H}^{Min}$) can be estimated based on Equation (20). As shown in Figure 8c, AT30P and AT15P exhibit higher strength recovery rates than the other composites, resulting in TiC oxidation at low temperatures and less time for complete crack healing and strength recovery (Figure 6). Further, it is worth mentioning that the $\bar{R_{s}}$ of AS30P0.2MnO is at 1273 K, which is almost the same as that of AT15P, although the values of $k_{p}$ of the SiC-healing agent (MnO) are lower than those of the TiC-healing agent. This difference is due to the fact that the values of ∆ρ for the SiC-healing agent are smaller than those of the TiC-healing agent, suggesting the importance of healing product selection for self-healing ceramics design. In addition, the data plot is linear, approximately below 1073 K. Meanwhile, for the SiC-type healing agents, including AS15P, AS30P, MS20P, and AS30P0.2MnO, the data plots are nonlinear because of the crack closure by residual stress release approximately above 1073 K as described by Equation (11). Furthermore, the $\bar{R_{s}}$ for the SiC-type healing agent oxidation follows the order of AS30P0.2MnO >> MS20P > AS30P > AS15P, suggesting that the selection of the healing activator and matrix together with the healing agent is important for designing ceramics that can realize rapid self-healing of surface cracks.

In this way, we demonstrated that the proposed framework enables the self-healing ability of all composites to be assessed by using only the limited data set of strength recovery. Notably, the effects of both the AE and FF on the oxidation rate, volume-gain rate, crack-gap filling, and healing rate corresponding to the temperature and time can be reasonably described by the universal kinetics model of strength recovery. Furthermore, the kinetic parameters AE and FF of self-healing ceramic composites, which are necessary for the examination of practical applications, can be determined using the proposed framework.

## 6. Conclusions

This article proposed a novel framework for determining the kinetic parameters of self-healing ceramic composites using experimental strength recovery data, micro-structural features, and exact experimental conditions by adopting a universal kinetics model of strength recovery [39]. The proposed method can reasonably explore kinetic parameters, and its effectiveness was demonstrated by comparing the experimental and theoretical strength recovery behaviors under several healing conditions. TiC healing agents have higher oxidation capabilities than SiC and MAX-phase healing agents, even at low temperatures, as can be confirmed by the AE and FF; both parameters play significant roles in rapid strength recovery, depending on the temperature conditions and time during service.

Note that the proposed framework, based on the universal kinetics model, can be applied to estimate the kinetic parameters not only for commonly used healing agents, such as SiC, TiC, and Ti_2_AlC, but also other healing agents. Moreover, the obtained parameters can be used for finite element analysis of self-healing under arbitrary environmental conditions [38]. Collectively, the proposed method will be helpful in selecting healing agents and matrices for the design of next-generation self-healing ceramic composites used in high-temperature applications. To further refine the parameter determination method, the super healing phenomenon and recovery of the fracture toughness of self-healing ceramics owing to healing should be examined in the future.

## Figures and Tables

**Table 1:** Different self-healing ceramic composites, along with damage formation methods, healing conditions, and bending strength test methods. Al_2_O_3_, mullite, Si_3_N_4_, ZrO_2_, and Ti_2_AlC are the matrix of material composition. (p) and (w) represent the healing agent’s particle and whisker. Experimental strength recovery behaviors, healing conditions, and micro-structural features on fractured surfaces of AS15P [40], AS30P [39], AS30P0.2MnO [41], MS20P [42], AT15P [43], AT30P [43], AMAX20P [44], and composites are considered for this study.

Material Composition					Damage Formation			Healing Conditions					Test Method	Ref.
Matrix	Healing Agent	Volume Fraction [%]	Healing Activator	Sintering Additives	Indentation Method	Crack Length [µm]	Aspect Ratio	Heating Rate [°C/min]	Cooling Rate [°C/min]	Holding Temperature [K]	Holding Time [hr.]	Environment		
Al_2_O_3_	SiC (p)	30	---	---	Vickers	50~600	0.9	10 °C/min	5 °C/min	973~1573	0.0167~1000	Air	3-point bending	[39]
	SiC (p)	30	MnO	---	Vickers	110	0.9	10 °C/min	5 °C/min	1273	0.018~0.15	Air	3-point bending	[41]
	SiC (p)	15	---	---	Vickers	100	0.9	10 °C/min	5 °C/min	1173~1673	1~300	Air	3-point bending	[40]
	TiC (p)	15~30	---	---	Vickers	110	0.9	10 °C/min	natural	673~1073	1	Air	3-point bending	[43]
	Ti_2_AlC (p)	20	---	---	Vickers	80	0.9	10 °C/min	100 °C/min	1073~1273	0.25~16	Air	4-point bending	[44]
	SiC (p)	15	---	---	Vickers	100	0.9	10 °C/min	10 °C/min	1273~1773	0.166~50	Air	3-point bending	[15]
	SiC (p)	15	---	---	Vickers	70~340	---	---	---	1273~1573	1	Air	3- & 4-point bending	[16]
	SiC (p)	15	---	---	Vickers	100	0.9	10 °C/min	10 °C/min	1273~1723	1	Air	3-point bending	[17]
	TiC (p)	26.85	---	MgO & $TiB_{2}$	Vickers	300~600	---	8 °C/min	8 °C/min	873~1073	0~1	Air	3-point bending	[19]
	SiC (w)	20	---	---	Vickers	100	0.9	10 °C/min	5 °C/min	1473~1573	1~2	Air	3-point bending	[23]
	SiC (p)/ Nano Ni	6	---	---	Vickers	40~80	---	---	---	1273~1573	1~48	Air	3-point bending	[21]
	Nano-Ni	5	---	---	Vickers	200	---	---	---	873~1473	1~6	Air	3-point bending	[22]
Mullite	SiC (p)	20	---	---	Vickers	100~200	0.8~0.9	10 °C/min	10 °C/min	1273~1573	1~20	Air	4-point bending	[42]
	SiC (p)	15	---	Y_2_O_3_	Vickers	100~220	0.9	10 °C/min	5 °C/min	1273~1573	1	Air	3-point bending	[28]
	SiC (p)	15	---	Y_2_O_3_	Vickers	100	0.9	---	---	1473~1623	1	Air	3-point bending	[29]
Si_3_N_4_	SiC (p)	20	---	Y_2_O_3_	Vickers	100	0.9	---	---	1473	1~5	$N_{2}-O_{2}$	3-point bending	[24]
	SiC (w)	20	---	Y_2_O_3_	Vickers	200~1200	0.9	10 °C/min	10°C/min	1273~1573	1	Air	3-point bending	[25]
ZrO_2_	SiC (p)	10	---	Y_2_O_3_	Vickers	100	0.9	---	---	1073~1173	1~10	Air	3-point bending	[30]
Ti_2_AlC	---	---	---	---	Knoop	---	---	---	---	1273~1673	2	Air	3-point bending	[34]

## Data Availability

Not applicable.
